# Characterization of monoclonal antibodies against foot-and-mouth disease virus serotype O and application in identification of antigenic variation in relation to vaccine strain selection

**DOI:** 10.1186/1743-422X-11-136

**Published:** 2014-08-01

**Authors:** Ming Yang, Wanhong Xu, Melissa Goolia, Zhidong Zhang

**Affiliations:** 1 National Centre for Foreign Animal Disease, 1015 Arlington Street, Winnipeg R3E 3 M4, Manitoba, Canada

**Keywords:** Foot-and-mouth disease, Monoclonal antibody, Antigenic site, Vaccine matching

## Abstract

**Background:**

Foot-and-mouth disease (FMD) has severe implications for animal farming which leads to considerable financial losses because of its rapid spread, high morbidity and loss of productivity. For these reasons, the use of vaccine is often favoured to prevent and control FMD. Selection of the proper vaccine is extremely difficult because of the antigenic variation within FMDV serotypes. The aim of the current study was to produce a panel of mAbs and use it for the characterization of new isolates of FMDV serotype O.

**Results:**

A panel of FMDV/O specific mAb was produced. The generated mAbs were then characterized using the peptide array and mAb resistant mutant selection. Seven out of the nine mAbs reacted with five known antigenic sites, thus the other two mAbs against non-neutralizing sites were identified. The mAbs were then evaluated by antigenic ELISA for the detection of forty-six FMDV serotype O isolates representing seven of ten known topotypes. Isolates ECU/4/10 and HKN/2/11 demonstrated the highest antigenic variation compared to the others. Furthermore, the panel of mAbs was used in vaccine matching by antigenic profiling ELISA with O1/Manisa as the reference strain. However, there was no correlation between vaccine matching by antigenic ELISA and the gold standard method, virus neutralisation test (VNT), for the forty-six FMDV/O isolates. Nine isolates had particularly poor correlation with the reference vaccine strain as revealed by the low r_1_ values in VNT. The amino acid sequences of the outer capsid proteins for these nine isolates were analyzed and compared with the vaccine strain O1/Manisa. The isolate ECU/4/10 displayed three unique amino acid substitutions around the antigenic sites 1, 3 and 4.

**Conclusions:**

The panel of mAbs is useful to monitor the emergence of antigenically different strains and determination of relevant antigenic site differences. However, for vaccine matching VNT remains the preferred method but a combination of VNT, antigenic profiling with a panel of mAbs and genetic sequencing would probably be more ideal for full characterization of any new outbreak isolates as well as for selection of vaccine strains from FMDV antigen banks.

## Introduction

Foot-and-mouth disease (FMD) is a highly infectious and acute disease that affects cloven-hoofed animals such as cattle, pigs, goats, sheep and deer. Its rapid spread, high morbidity and loss of productivity have severe implications for animal farming which leads to considerable financial losses. For these reasons, the use of vaccine is often favoured to prevent and control FMD. Vaccination was used successfully to help control the FMD outbreak in the Netherlands in 2001 [1]. Vaccination may be an economically optimal strategy, although the questions of where, how, and when to use vaccination for FMD need to be further addressed [2].

Foot-and-mouth disease virus (FMDV) comes in seven serotypes (O, A, C, Asia 1, SAT 1, 2 and 3). Among the seven serotypes of FMDV, O and A are the most widespread. FMD viruses frequently change at different antigenic sites. Within the serotypes, there is considerable antigenic variability [3]. There is no cross-immunity among the seven serotypes. This is evidenced in animals that have previously been infected with one serotype, but remain susceptible to the six other serotypes. Consequently, FMDV-specific antibodies protect only against homologous, but not heterologous FMD outbreaks. Thus, the vaccine selected must be highly specific to the strain involved and matched as closely as possible with the outbreak isolate. It has been indicated that lack of vaccine-induced protection may involve the use of an inadequately matched vaccine [4].

A direct relationship has been shown between the level of serum neutralizing antibody and animal protection [5]. However, selection of the proper vaccine is extremely difficult because of the antigenic variation within FMDV serotypes. In general, methods for vaccine strain selection mainly rely on two in vitro indirect serological methods: (a) virus neutralisation test (VNT) using vaccine strain-specific serum pool [6] and (b) an ELISA using polyclonal antibodies [7]. VNT is more relevant to in vivo protection than other measures [8] and seems to produce the most reproducible inter-laboratory results [9]. Although the neutralisation test has been widely used for many years, it is time consuming and requires live virus. In addition, the results are inconsistent because of (1) different cells and different sera used and (2) different interpretation of cytopathic effect (CPE) in different laboratories. ELISA, on the other hand, has advantages over VNT because it is rapid and no live virus is required. But the ELISA using polyclonal antibodies is difficult to standardize.

Sequence analysis can reveal genetic changes of viruses. Thus it can reveal the emergence of new strains and may indicate if an outbreak isolate is similar to a vaccine strain [10]. However, the procedure is complicated and takes days to complete.

Antigenic profiling ELISA using mAbs provides a fast and more sensitive method for the characterization of field and vaccine strains [11-15]. A rapid and simple method to compare antigenic profiles and characterization of new field isolates has been reported using panels of mAb [16]. However, antibody binding sites were not well-characterized and identified in that study. Thus it is impossible to locate mutations and identify differences among isolates. Mahapatra et al. [17] reported that they were unable to find a correlation between the micro neutralization results and antigenic profiling ELISA using mAbs.

Up until now, the information is limited regarding the relationship of the r_1_ values in 2-dimentional (2D)-VNT, amino acid mutation on capsid protein using genetic sequencing and antigenic profiling using a well-characterized mAb panel. To achieve the goal of simplicity and speed up the vaccine matching process, a panel of mAbs against FMDV serotype O was produced. The epitopes recognized by these mAbs were characterized. This panel of mAbs was used in antigenic profiling ELISA. Forty-six FMDV/O isolates were examined using 2D-VNT and antigenic profiling ELISA. Nine isolates lacking close antigenic relationship with a vaccine strain O1/Manisa in VNT were further examined using genetic sequence analysis.

## Results

### Production of monoclonal antibody

A panel of FMDV/O specific mAbs were produced. Four groups of mice were inoculated separately with serotype O antigens (Campos, BFS, recombinant Capmpos/Brazil/58/VP1 or VP2 [18]. Fusions for each group of mice were performed and allowed for the production of FMDV/O specific hybridomas. After subcloning, the mAbs were designated and their isotypes characterized. Nine mAbs were selected and used in this study (Table 1). The mAbs’ reactivity and specificity against different FMDV serotypes were examined using FMDV serotype specific double antibody sandwich (DAS) ELISAs [18]. The results indicate that all mAbs are FMDV/O specific without cross reactivity against FMDV other serotypes (A, C, Asia 1, SAT1, 2, and 3) and other vesicular disease viruses (Swine vesicular disease and Vesicular stomatitis). Six of nine mAbs demonstrated virus neutralization activity. Three of them were non-neutralizing mAbs.

**Table 1:** Characteristics of monoclonal antibodies against FMDV/O and their binding sites

**Clone Name**	**Isotype**	**Immunization Ag**	**VNT results**	**Reactivity to recombinant protein**	**Antigenic Sites**	**Binding sites**
F1140O2-5	IgG1/k	O1 Campos	-	-	-	VP3 V^73^
F11VP2O-2	IgG1/k	Rec VP2	-	VP2	-	VP2 ^133^QK^134^
F12VP1O-2	IgG1/k	Rec VP1	-	VP1	Site1b	VP1 ^198^EARHKQKIVAPVKQTL^213^
F21-48	IgG2a/k	O1 BFS	+	VP1	Site1a,5	VP1 ^148^ L
F21-64	IgG2a/k	O1 BFS	+	VP1	Site1a,5	VP1 ^136^YSRNAVPNLRGDLQVL^151^
F21-34	IgG2b/k	O1 BFS	+	-	Near site2	VP2 ^68^D
F21-58	IgG2b/k	O1 BFS	+	-	Site2	VP2 ^77^R
F21-41	IgG2b/k	O1 BFS	+	-	Site3	VP1 ^43^TP^44^
F21-18	IgG2a/k	O1 BFS	+	-	Near site4	VP3 ^59^G

-: Negative result; +: Positive result.

### Identification of mAbs’ binding epitope

**Figure 1:**
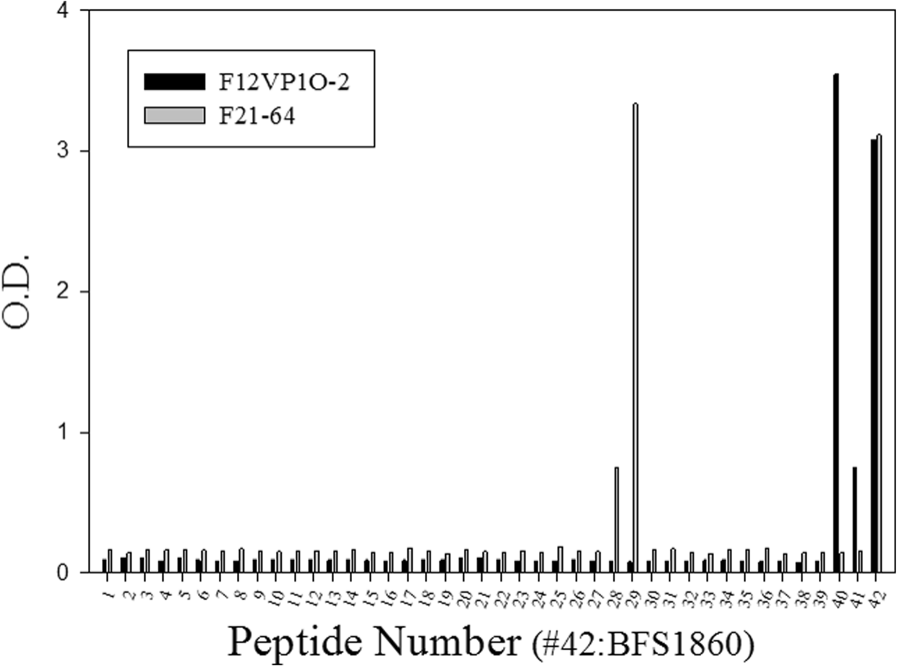
**Reactivity of mAbs (F12VP1O-2 and F21-64) with forty-one O/VP1 overlapping peptides in an indirect ELISA.** Forty-one overlapping peptides and purified FMV/O were coated onto 96-well plate. The reactivities of the mAbs to the peptides and O1/BFS were detected with HRP anti-mouse IgG, followed by a substrate.

In order to define the binding epitopes of the mAbs, the reactivity of the mAbs against recombinant VP1 and VP2 was examined using an indirect ELISA. Four mAbs reacted with the recombinant proteins (Table 1). The result showed that three mAbs (F12VP1O-2, F21-48 and F21-64) reacted with recombinant VP1 protein, while F11VP2O-2 reacted with recombinant VP2 protein (Table 1). This confirmed that the epitopes recognized by these four mAbs are linear, because the recombinant proteins were expressed in E. coli. The other mAbs failed to react with the recombinant proteins suggesting that the epitope recognized by these mAbs are conformational which is dependent on the integrity of viral particle structures. To further locate mAb binding epitopes, 41 peptides representing FMDV/O/VP1 and 42 peptides corresponding to O/VP2 were synthesized. Reactivity of the mAbs against peptides was examined using a peptide ELISA (Figure 1). The mAb F21-64 reacted with peptides 28-29 corresponding to the GH loop of VP1 at amino acids 136-151 (YSRNAVPNLRGDLQVL) which was recognized as the antigenic site1a. The mAb, F12VP1O-2 reacted with peptides 40-41 corresponding to the C-terminal residues of VP1 at amino acids 198-213 (EARHKQKIVAPVKQTL). This region was identified as the antigenic site1b, despite the fact that the mAb F12VP1O-2 did not demonstrate virus neutralization activity. The mAb F21-48 and F11VP2O-2 failed to react with any VP1/VP2 peptides, although it reacted with recombinant proteins.

### Monoclonal antibody resistant mutant selection

Since conformation-dependent and certain linear epitopes could not be identified using the peptide array method, mAb resistant mutant selection was used for the five out of six neutralizing mAbs. The mAb F21-48 reacted with recombinant VP1, but failed to react with VP1 peptides. Thus its binding site was also identified using the mutant selection. In the mutant selection, the viruses were allowed to grow in the presence of the mAb with dilutions 100-fold lower than minimum neutralization titer. After six passages, six mutants were selected and analyzed using a DAS ELISA. ELISA results showed that the polyclonal serum reacted with all six parental viruses and selected mutants, whereas, the mAbs reacted with only parental viruses, not the matching mutants. The ELISA results indicated that the mAb binding sites were fully depleted in those selected mutants.

**Figure 2:**
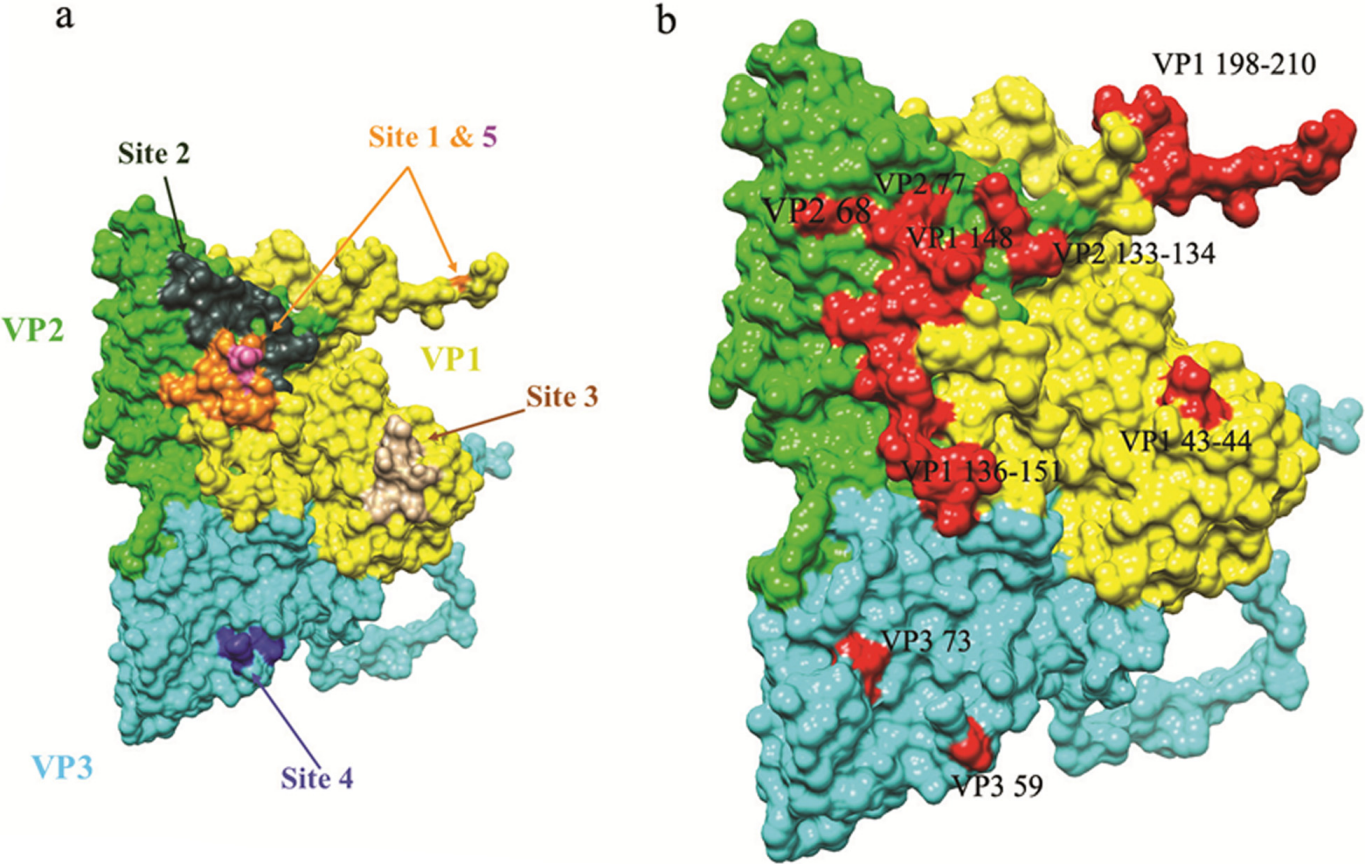
**Localization of antigenic sites in FMDV/O capsid proteins.** The O1/ BFS1860 crystal structure (PDB # 1FOD) was manipulated with Chimera and shown in surface format. **a**. Locations of previously identified 5 antigenic sites; **b**. Locations of identified epitopes of anti-FMDV/O mAbs (summarized in Table 1) showing in red. For VP1 residues 211-213: no corresponding structure in 1FOD (FMDV/O1/ BFS1860 crystal structure).

The five mutant sequences of P1 gene encoding capsid proteins (VP1, VP2, VP3 and VP4) were compared with parental O/BFS P1 gene. Mutants are named based on their matching mAbs. The sequence data revealed that two selected mutants with the mAbs F21-34 and F21-58 recognized antigenic site 2 in the region of VP2 amino acid positions about 68 and 77, respectively (Table 1). The mutant selected with mAbs F21-41 was found to recognize antigenic site 3 at VP1 amino acid positions close to 43-44, whereas the mutant selected with F21 -18 recognized near antigenic site 4 at amino acid position about 59 located in VP3. The mAb F21-48 recognized antigenic site1a on the GH loop of VP1 at amino acid position 148 (Table 1). The FMDV/O antigenic sites1a (site 5), 1b, 2, 3 and 4 identified in this study were similar as previously published [11,19,20]. The locations of mAb binding sites are shown in the FMDV 3D structure (Figure 2).

In addition, two mAbs F1140O2-5 and F11VP2O-2 against non-neutralizing sites were predicted to bind VP3 at amino acid position around 73, and VP2 region at amino acid 133-134 respectively, based on the reactivity of 46 isolates in antigenic ELISA and sequence analyses of P1 region (unpublished data).

### Two-dimensional virus neutralization

Antigenic matching is usually estimated indirectly, by in vitro analysis of the antibody response to vaccination and by comparing the cross-reactivity of sera collected from vaccinated animals against the vaccine and field virus. The 2D-VNT was performed to determine the antigenic relationship between the vaccine strains and field isolates. A 2D-VNT was performed using an antiserum (21 days post vaccination) raised against FMDV/O1/Manisa (WRLFMD). A total of forty-six isolates representing seven of ten known serotype O topotypes were examined and their r_1_ values determined as shown in Table 2. Thirty-seven isolates demonstrated r_1_ > 0.3, indicating that these isolates are antigenically similar to the reference strain, O1/Manisa. Nine viruses (AFG/41/11, ECU/4/10, ETH/39/09, HKN/2/11, IRN/11/06, KUW/1/11, TAW/12/98, UAE 9/09, and VIT/32/11) had r_1_ values of less than 0.3 indicates that these strains are not sufficiently related and cross-protection is less likely to occur, suggesting that significant antigenic variations exist between reference strain and these field isolates. Our data are consistent with those reported by WRLFMD (Table 2).

**Table 2:** List of FMDV/O isolates used in the study

**FMD/O Isolates**	**GenBank accession#**	**Topotype**^**a**^	**Antigenic ELISA r**^**b **^**value**	**VNT r**_**1 **_**by NCFAD**^**c**^	**VNT r**_**1 **_**by WRLFMD (reported year)**
Manisa		ME-SA	1.00	1.00	-^d^
AFG/41/11	KJ606977	ME-SA	0.69	0.13	< 0.3 (2011)
BHU/39/04		ME-SA	0.45	0.63	-
BUL/3/11		ME-SA	0.78	2.63	> 0.3 (2011)
IRAQ/5/94		ME-SA	0.70	0.52	-
IRN/11/06	KJ606980	ME-SA	0.41	0.21	-
IRN/31/10		ME-SA	0.68	0.39	-
IRN/8/05		ME-SA	0.39	0.63	-
KRG/1/06		ME-SA	-0.13	1.66	-
KRG/2/06		ME-SA	-0.28	2.34	-
KUW/1/11	KJ606981	ME-SA	0.71	0.16	<0.3 (2011)
EGY/8/06		ME-SA	0.81	0.66	-
NEP/3/10		ME-SA	0.47	1.17	> 0.3 (2011)
PAK/1/10		ME-SA	0.52	0.79	0.46 (2010)
SAU/1/09		ME-SA	0.68	0.79	0.74 (2009)
SAU/4/05		ME-SA	0.57	0.45	0.78 (2005)
SAU/7/08		ME-SA	0.61	0.85	-
UAE/2/03		ME-SA	0.76	0.87	-
UAE/2/10		ME-SA	0.57	1.32	0.39 (2010)
UAE/9/09	KJ606983	ME-SA	0.68	0.12	-
UKG/11/01		ME-SA	0.65	1.45	-
UKG/13708/01		ME-SA	0.75	1.32	-
UKG/14221/01		ME-SA	0.76	1.00	-
VIT/32/11	KJ606984	ME-SA	0.34	0.13	<0.3 (2011)
KEN/62/09		EA-1	0.66	0.39	-
ETH/39/09	KJ606978	EA-3	0.48	0.02	0.16 (2009)
NIG/15/09		EA-3	0.30	1.58	> 0.3 (2010)
SOM/1/07		EA-3	0.53	0.81	-
SUD/3/08		EA-3	0.55	0.79	-
TAN/5/09		EA-2	0.70	2.69	> 0.3 (2010)
ZAM/1/10		EA-2	0.64	1.00	-
BFS1860		Euro-SA	0.44	0.32	-
ECU/4/10	KC519630	Euro-SA	0.18	0.05	-
UKG/685/07		Euro-SA	0.74	2.00	-
HKN/1/10		SEA	0.58	0.63	0.5 (2010)
HKN/2/11	KJ606979	SEA	0.07	0.10	<0.3 (2011)
MAY/1/05		SEA	0.59	0.78	-
MYA/1/10		SEA	0.50	1.70	-
MYA/11/09		SEA	0.55	1.55	-
SKR/4/10		SEA	0.58	1.20	0.57 (2010)
VIT/7/06		SEA	0.66	0.81	-
SEN/8/06		WA	0.48	0.60	-
MAI/15/06		WA	0.44	1.05	-
TAW/10/97		CATHAY	0.73	0.35	-
TAW/12/98	KJ606982	CATHAY	0.76	0.19	-
VIT/9/05		CATHAY	0.69	0.50	-

^a^Middle East-South Asia (ME-SA); Southeast Asia (SEA); East Africa (EA); Europe-South America (Euro-SA); West Africa (WA).

^b^Correlation coefficient between O1Manisa and each isolate.

^c^National Centre for Foreign Animal Disease (NCFAD), Canada.

^d^Information is not available.

The correlation coefficient (r) for the panel of mAb was calculated between O1/Manisa and each isolate in the antigenic ELISA and the r_1_ values was determined in 2D-VNT using an O/Manisa vaccinated serum.

### Antigenic profiling ELISA using the mAb panel

The panel of well characterized mAbs were used in the antigenic profiling ELISA. To standardize the amount of virus captured to the plate in the antigenic profiling ELISA, a serotype independent mAb (F21-42) was used as a control antibody instead of polyclonal serum. This mAb demonstrated a consistent binding to all virus isolates [18], while the polyclonal anti-FMDV/O mouse serum pool demonstrated poor binding to 6 out of 46 isolates. The relativities of the mAb panel to the nine isolates demonstrated poor relationship with the vaccine strain O/Manisa in 2D-VNT were examined closely. Two isolates ECU/4/10 and HKN/2/11 showed the highest antigenic variation among the nine isolates in the antigenic profiling ELISA (Table 3). Four of the nine mAbs failed to react with these two isolates (Rx < 0.2). In addition, one mAb demonstrated low reactivity (Rx < 0.5) to HKN2/11 and two mAbs demonstrated low reactivity to ECU4/10. It is assumed that some mutations occurred on the antigenic site 1, 2 and 4, as well as a non-neutralizing site for each isolate. This explains why these two isolates demonstrated very low r_1_ values in 2D-VNT.

**Table 3:** **Reactivity of the mAb panel against FMDV/O nine isolates with r**_**1 **_**value <0.3 in 2D-VNT**

**Viruses**	**F1140O2-5 VP3**	**F11VP2O-2 VP2**	**F12VP1O-2 Site 1b**	**F21-48 Site 1a,5**	**F21-64 Site 1a,5**	**F21-34 Site 2**	**F21-58 Site 2**	**F21-41 Site 3**	**F21-18 Near site 4**
ECU/4/2010	0.10	1.07	0.49	1.42	0.45	-0.004	0.014	1.76	0.014
HKN/2/2011	1.21	-0.02	1.12	0.25	0.98	0.05	0.02	1.36	0.05
VIT/32/11	1.22	1.02	0.56	1.51	1.52	0.15	0.04	1.44	1.39
AFG/41/11	1.13	1.03	0.65	1.17	1.20	1.11	1.19	0.28	0.12
ETH/39/2009	1.20	1.05	0.39	1.2	1.2	1.15	1.19	1.10	1.05
UAE/9/2009	1.30	1.03	0.83	1.39	1.41	0.95	1.36	1.33	0.76
IRN/11/2006	1.07	1.02	0.49	1.13	1.10	0.99	1.10	1.08	1.06
TAW/12/98	1.58	0.80	0.06	1.57	1.63	1.55	1.61	0.07	-0.02
KUW/1/11	1.26	1.08	0.33	1.30	1.31	1.25	1.25	0.90	0.73

The values are corrected OD values (R_X_ in M& M) determined in the antigenic ELISA.

Values <0.2: no reactivity; <0.5 weak reactivity.

The antigenic site 1b identified by mAb F12VP1O-2 showed the highest variation compared with others. Five out of nine isolates showed littler to no reactivity (Rx < 0.5) with this mAb. The antigenic sites 2 and 4 demonstrated higher variations compared with sites1a and 3 based on the ELISA results. All mAbs reacted with the isolate UAE/9/2009 indicating that there is no significant change at these mAb binding sites.

For vaccine matching purpose, reactivity value (Rx) obtained with each mAb for every field isolate and the reference strain (O1/Manisa) was used to calculate r values to normalize the results [16,19], since the O1/Manisa mAb panel was not available. The r values (Table 2) were used to determine the strength of the linear association between the vaccine strain and field isolates. The correlation efficiency between the 2D-VNT and the antigenic ELISA were also determined based on the 2D-VNT r_1_ and antigenic profiling ELISA r values. A poor relationship between the 2D-VNT and the mAb antigenic profiling ELISA results was observed for the forty-six isolates with a correlation coefficient of r = 0.085, p = 0.57.

### Sequence analyses

Sequence analysis is used to determine whether an isolate is genetically similar to a vaccine strain. The P1 sequences encoding capsid proteins of 9 FMDV/O viruses with r_1_ < 0.3 were determined. Each contains 2208 nucleotides with the exception of UAE/9/09 and ECU/4/10, which have 2205 nucleotides. The deduced P1 polyprotein is 736 amino acids long but the P1 polyprotein of UAE/9/09 and ECU/4/10, both of which have an amino acid deletion within the VP1 G-H loop when aligned, is 735 amino acids long.

### Identification of antigenic site variation

**Figure 3:**
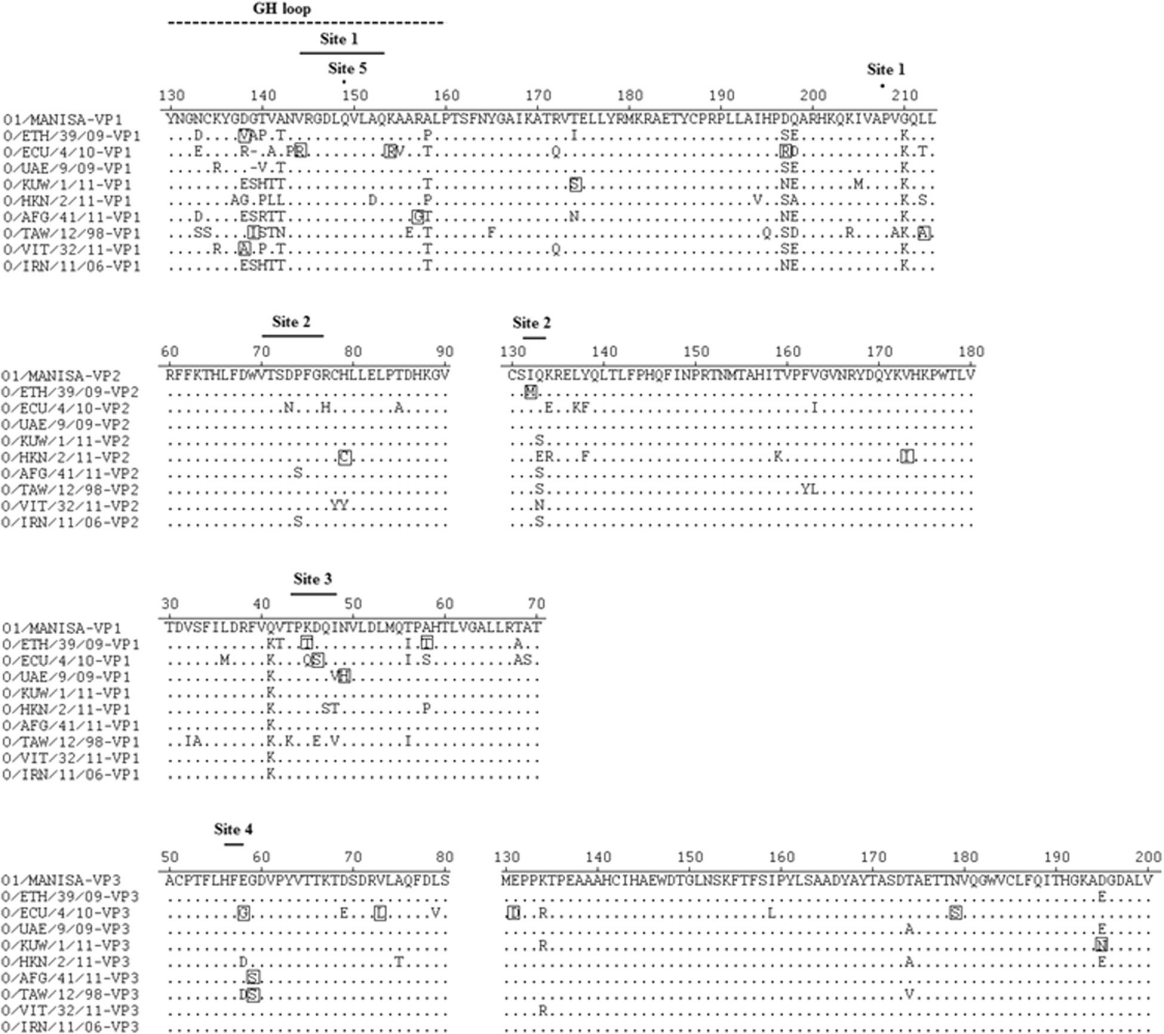
**Alignment of partial capsid amino acid sequences of unmatched FMDV/O field isolates and reference strain O1/Manisa.** The capsid proteins of VP1, VP2, and VP3 of the 9 FMDV isolates (shown in Table 3) were aligned by Clustal-W. Only the regions containing 5 previously determined antigenic sites and unique amino acid substitutions of unmatched isolates and reference strain are shown for clarity. The identical amino acid residues are indicated by dots. Amino acid deletions are indicated as hyphens. The single letter amino acid code is used. Previously identified antigenic sites for FMDV/O are indicated with solid horizontal lines or closed circles above the sequences. Amino acid residues with unique substitutions in the 9 sequences are indicated by open boxes, and the VP1 GH loop residues 130-160 is indicated by a dashed line shown above the sequences.

To better understand antigenic relations between the vaccine strain and field isolates at amino acid level, the amino acid sequences of outer capsid proteins were analyzed. Comparison of the five antigenic sites recognized by the mAb panel revealed that antigenic sites 2, 3 and 4 are identical for O1/BFS and O1/Manisa. There are two and six amino acids variations for antigenic site 1a and 1b recognized by the mAbs F12VP1O2-1 and F21-64, respectively for O1/BFS and O1/Manisa. The nine isolates with r_1_ < 0.3 in 2D-VNT were investigated and compared with the vaccine strain O1/Manisa. Figure 3 summarized amino acid alterations observed on the surface of outer capsid proteins in/near five known antigenic sites. In comparison with a vaccine strain O1/Manisa, analysis of 5 previously identified antigenic sites indicated that ECU/4/10 has the highest antigenic variation in or near antigenic sites. This isolate displays three unique amino acid substitutions around the neutralizing sites 1, 3 and 4. There are amino acid deletions at 139 VP1 GH loop and other amino acid substitutions located on VP1 and VP3. The high sequence variation of the ECU/4/10 explained the low r_1_ value in 2D-VNT (r_1_ = 0.05) and a very weak correlation coefficient (r = 0.184) in antigenic profiling ELISA (Table 2). The isolate ETH/39/09 has unique amino acid alterations located around the antigenic site 2 and 3. However, the binding of the mAb panel with ETH/39/09 failed to show any significant reduction at these locations. But a low binding was observed with the mAb (F12VP1O-2) located on VP1 C-terminal (Table 3).

All other isolates did not show significant amino acid alterations compared with the vaccine strain O1/Manisa at the antigenic sites. However, some amino acid variations were occurred near the antigenic sites. Within the GH loop of the VP1, it is notably a deletion at VP1 amino acid position 139 of UAE/9/09. This may explain low r_1_ values in 2D-VNT for this strain. Unique amino acid substitutions for TAW/12/98 (aa 212-A) and KUW/1/11 (aa 205-M) located at the antigenic site 1b were confirmed by sequence analysis. Unique amino acid changes were not found within the outer capsid proteins of the isolate IRN/11/2006.

## Discussion

The analysis of antigenic differences is critical for FMD surveillance and vaccine strain selection. Emergence of antigenically different strains in FMD outbreak needs to be determined in relation to the respective vaccine strains using appropriate techniques [21]. As neutralizing antibody titers correlate well with protection in animals, the virus neutralization test has been adopted since 1977 [6] and is widely used as the reference test system for vaccine strain selection. Although the 2D-VNT has been considered the gold standard, significant variation has been described with the VNT results [6,22]. Mattion [9] indicated that r_1_ value estimations using low serum titer become less precise. Therefore depending on only a single test for vaccine matching may not be suitable.

To better antigenically monitor new outbreak strains, a panel of FMDV/O specific mAbs was produced. Seven mAbs that reacted with the neutralizing sites and two mAbs that reacted with the non-neutralizing sites were included in this panel. Four mAbs reacted with recombinant proteins indicating binding sites of these mAbs were linear. The binding sites for mAb F12VP1O-2 and F21-64 identified using peptide ELISA are located on VP1 antigenic site 1b (aa198-213) and 1a (aa136-151). Although the mAb F12VP1O-2 binding site is located in the antigenic site 1b, it was unable to neutralize the virus in a VNT. A possible explanation may be that site 1a (the βG ± βH loop) and 1b (the carboxy terminus) on VP1 together involve antigenicity and receptor binding [23]. The mAb F12VP1O-2 against only C-terminal would not be able to completely block virus attachment, thus demonstrating negative neutralizing activity.

In general, linear antibody binding epitopes can be located using overlapped peptides. Surprisingly, F11VP2O-2 and F21-48 failed to react with any peptides, even though they reacted with recombinant O/VP 1 and VP2 in an indirect ELISA. A similar observation has been reported previously [24]. An explanation is that mAb reactivity with a continuous epitope not only depends on the amino acid sequence, but also on a stretch of contiguous residues to assume the correct conformation [25]. The binding sites of the other neutralizing mAbs with conformational epitopes were identified using the mAb resistant mutant selection method. Though the mAb F21-48 reacted with a linear epitope, but it was unable to react with any VP1 peptides. The exact binding site was also identified using the mutant selection method. After the selection in the presence of high concentrations of mAbs, the neutralizing mAb resistant mutants were generated and genetically analysed. The nucleotide mutations causing one significant amino acid substitution in protein structures were determined. The substituted amino acid residue was considered the antigenic site recognized by the mAbs. Combining the results obtained from the peptide ELISA and the mutant selection method, we concluded that the binding sites of seven mAbs are located on all five previously identified antigenic sites on FMDV/O involving VP1, VP2, and VP3 major capsid proteins [11,19,26,27]. It was confirmed that these sites were exposed in the 3D model of protein structure.

Using this well characterized mAb panel, an antigenic profiling ELISA was developed with a modification from previously reported ELISA [15]. To correct the amount of virus trapped by the capture antibody, a serotype independent mAb (F21-42) [18] was used to standardize the ELISA results instead of a polyclonal serum. This mAb demonstrated a consistent reactivity to all virus isolates, while the polyclonal anti-FMDV/O mouse serum pool demonstrated poor binding to some isolates.

The forty-six FMDV/O field isolates were analysed using the mAb antigenic ELISA. The isolates ECU/4/10 and HKN/2/11demonstrated highest antigenic variation compared with other isolates. Four of the nine mAbs failed to react with these two isolates. In addition, one mAb demonstrated low reactivity to HKN2/11 and two mAbs demonstrated low reactivity to ECU4/10. It is assumed that various amino acid alterations occurred in/near the antibody binding sites. The mutated binding sites identified by the mAbs for ECU/4/10 are located on the antigenic sites 1a, 1b, 2, 4 and one on non-neutralizing site. This result of high antigenic variation is consistent with previously reported data for the ECU/4/10 isolates [28]. Viruses circulating in Ecuador during the years 2009–2010 were examined using monoclonal antibody profiling. The results showed that the viruses lost reactivity with the four mAbs, three of them with neutralizing properties. Moreover, results obtained with in vivo challenge indicated a lack of protection by the vaccine virus (O1/Campos) [28].

Both the GH loop and the C-terminus of VP1 formed antigenic site1 (a, b) are highly exposed regions on virus surface [23]. It has been indicated that the region of VP1^200-273^ is important to both antigenicity and receptor binding of FMDV [19,29-31]. It is involved in cell attachment because selective removal of these residues resulted in FMDV particles no longer capable of binding to cells [32]. In this mAb panel, a mAb F12VP1O-2 is able to recognize this region located on the C-terminal of VP1^198-213^. Five out of nine isolates demonstrated weak to no reactivity to this mAb indicating high variations at this mAb binding site. Unique amino acid substitutions for TAW/12/98 (aa 212-A) and KUW/1/11 (aa 205-M) were confirmed by sequence analysis. Those substituted amino acids are adjacent to the antigenic site 1b (VP1^208^) and might explain a low r_1_ value in 2D-VNT. Likely, this mAb can be used to monitor whether any mutation occurred in this location.

Correlation coefficients were used to determine the relationship between the 2D-VNT and the antigenic profiling ELISA. However, in current study, a poor relationship for 46 isolates between the two tests was observed (r = 0.085, p = 0.57). In this study, the 2D-VNT was carried out using a polyclonal serum obtained from the O1/Manisa vaccinated cattle. Whereas the panel of mAbs used in the antigenic ELISA was raised against the O1/BSF (O1/Campos) strain. A previous report indicated that O1/Manisa and O1/Campos strains are related but they were not a perfect match by the 2D-VNT (r_1_ = 0.64/0.62) [33]. The sequence analysis revealed that the five antigenic sites identified by the mAb panel in O1/BSF are identical to O1/Manisa in antigenic sites 2, 3 and 4, but some amino acids changes were observed in site 1. To obtain better results for vaccine matching, mAbs raised against each specific vaccine strain should be used. In practice, this is impossible because of the large amount of work required for mAb production and characterization. Alternatively, a pooled of polyclonal sera from O1/BFS vaccinated animals should be used in the 2D-VNT to ensure the results are comparable; however, we were unable to obtain the pooled O1/BFS vaccinated polyclonal sera. In spite of this, it is unlikely that the poor correlation between the 2D-VNT and the antigenic ELISA is due to the fact that different strains were used to generate the detecting antibodies in each assay for two reasons. Firstly, despite the fact that the mAbs were raised against O1/BFS antigens, the locations of antigenic sites are common for all serotype O isolates. Thus any mutations that occur in these sites should be detectable using the mAb panel. Secondly, the r values of antigenic profiling ELISA for each isolate were calculated versus the reference strain (O1/Manisa). Similar finding was reported by Mahapatra et al., [17] who used the antibodies against the same strain for mAb antigenic ELISA and VNT; they also observed a negative correlation between the two tests.

A possible explanation for the poor relationship between the 2D-VNT and the antigenic ELISA may be that mAbs are a monovalent antibodies and that a single mutation occurring in the binding site may cause poor mAb binding, but may not affect 2D-VNT results by polyclonal antibodies. Another possibility could be that in addition to the known sites, other undefined sites (neutralizing or non-neutralizing) may also be important in the induction of a protective immune response. This was supported by the fact that animals with low levels of neutralizing antibodies could also be protected [34-37]. A broad repertoire of epitope specificities following vaccination has been observed in previous studies [38,39]. Also, Nagendrakumar [33] have reported that the hyperimmune sera collected from vaccinated animals were unable to compete with the panel of mAbs used in their study.

Current studies have shown that the antigenic profiling ELISA is not a substitute for the 2D-VNT in vaccine matching because of the poor correlation between the two tests. However, it may still be used as one of the parameters to measure how field isolates are antigenically related to a vaccine strain and can provide valuable information on why a vaccine strain and a field isolate do not match. A good example is that this approach has been used successfully to define the epitope mutations of ECU/4/10, although approximately a 10% sequence difference in VP1 with the vaccine strain O1/Campos has been reported [28]. Our finding explained previous finding that why there are no matching vaccine strains for this isolate.

To improve the current approach, more mAb representative of each antigenic site and multi panels of well-defined mAbs against different strains should be included and used in antigenic ELISAs. More non-neutralizing mAbs should also be included in the panel since non-neutralizing antibodies might also contribute to protection [40,41]. These will allow the antigenic ELISA results to be more compatible with the 2D-VNT.

A powerful screening method for characterising FMD viruses is the genetic sequencing of the capsid protein. The genetic sequence can reveal either emergence of new strains or how similar outbreak isolates are to vaccine strains. In order to see whether any association exists between amino acid variation and pattern of r_1_ value in 2D-VNT, deduced amino acids in the P1 region of 9 isolates with r_1_ < 0.3 were compared with a vaccine strain O1/Manisa. The isolate ECU/4/10 and ETH/39/09 display multiple unique amino acid substitutions and amino acid depletion on several neutralizing sites. The high sequence variation of these two isolates explained the low r_1_ value for 2D-VNT. All other isolates did not show significant amino acid alterations compared with the vaccine strain O1/Manisa on the antigenic sites. However, amino acid substitutions near antigenic sites were observed. The changed amino acids near antigenic sites may alter the structure of outer capsid proteins, rendering antibodies unable to bind to viruses and preventing reference serum from neutralizing the viruses.

It has been emphasized that the neutralizing site 1 (VP1^140-160^) plays a role in the antigenicity of FMDV. However the residue at position 139 located in the immunodominant region of VP1 (βG-βH loop) is also highly variable [42]. Amino acid modification at position 139 contributes to serum neutralization resistance in a serotype O isolate [43]. In the current study, four isolates, ETH/39/09, UAE 9/09, TAW/12/98 and VIT/32/11 showed amino acid substitution or deletion at position 139 of VP1 which may explain low r_1_ values in 2D-VNT. Similarly, Das et al. [42] noted that three isolates had an amino acid deletion at position 139 of VP1. This finding confirmed that this position (VP1^139^) is antigenically important. In future sequence analysis, attention should be paid to this region. Substitutions outside of the antigenic sites have been shown to play an important role in the antigenic diversification of FMDV [20,44]. Although one strain (IRN 11/2006) demonstrated a low r_1_ (0.21) value in 2D-VNT, no unique amino acid changes were identified in/near any neutralizing site of the outer capsid proteins. A possible reason for the low r_1_ value in 2D-VNT might be that the polyclonal serum used in the 2D-VNT is not a serum pool. It has been suggested that a suitable reference serum for vaccine matching r_1_-value experiments should be a pool or a medium to high titer serum [9]. The r_1_ value was higher than 0.3 when a different O1/Manisa vaccinated serum (National Centre for Foreign Animal Disease, Canadian Food Inspection Agency) was used in the 2D-VNT.

In comparison to the results of antigenic mAb ELISA and sequence analysis, multiple mAb binding reduction (sites 1, 2, 4) in ELISA and several amino acid alterations in the antigenic sites (sites 1, 3, 4) by sequence analysis were observed for isolate ECU4/10. No close relationship was observed between mAb binding and variations in antigenically critical residues for other isolates. Some unique amino acid changes located in/near antigenic sites were identified on virus capsid structural proteins by sequence analysis. On the contrary, the mAb reactivity with those isolates did not show any significant reduction. The isolate ETH 39/2009 demonstrated high antigenic variation by sequence analysis, but only one mAb binding reduction was detected. One possible explanation may be that although the amino acid mutation occurred in/near antigenic sites, residues are not located in the central part of epitopes. Thus the binding affinity is not affected significantly. Experiments of antibody-antigen binding revealed that the amino acids in the central part of the epitope–antibody site make the majority of the contribution to the antibody–antigen interaction [45]. A second possible explanation may be that despite the amino acid changing, the protein still folded into the similar 3D capsid structure. Thus the antibody binding was not reduced.

## Conclusions

In the present study, a panel of mAbs against FMDV/O was produced and characterized. The seven mAbs reacted with all 5 antigenic sites and the other two mAbs recognized non-neutralizing sites. The panel of mAbs is useful to monitor and define the emergence of antigenically different strains giving an approximation of antigenic differences and to help study the nature of the evolution of antigenic variation. The antigenic ELISA is the most rapid test and takes only a short time to obtain results. Sequence analysis combined with antigenic sites identification on capsid regions can reveal existence of amino acid substitution or deletion of field isolates. Both methods are valuable tools to assist in the profiling of outbreak strains in relation to the respective vaccine strains. However, for vaccine matching VNT remains the preferred method but a combination of VNT, antigenic profiling with a panel of mAbs and genetic sequencing would probably be more ideal for full characterization of any new outbreak isolates as well as for selection of vaccine strains from FMDV antigen banks.

## Material and methods

### Preparation of FMDV

All of the FMDV isolates used were obtained from the World Reference Laboratory for FMD (WRLFMD) at the Pirbright Institute (Pirbright, UK). FMD viruses were amplified in Mengeling-Vaughn Porcine Kidney (MVPK) cells [46]. The cells were cultured in Alpha Modification of Eagle’s medium (AMEM; WISENT Inc. Canada) supplemented with 10% fetal bovine serum (FBS) and 2 mM L-glutamine. The culture supernatants were clarified by centrifugation at 8000 rpm for 30 minutes. The procedure for virus 2-Bromoethylamine Hydrobromide (BEI) inactivation and purification were performed as previously described [18]. The inactivated and purified FMDV was used for mice inoculation.

### Production of monoclonal antibodies

Mice immunizations and mAb production were performed as previously published [18]. Briefly, four groups of female BALB/C mice were inoculated subcutaneously with each serotype/O antigen, inactivated and purified Campos, BFS, and recombinant Capmpos/Brazil/58/VP1 and VP2 [18] in separate immunizations. The antigens were mixed in an equal volume of TiterMax Gold (TiterMax USA Inc., Norcross, USA). Two to three identical boosts were administered at four week intervals for each group. Mice were boosted with the same antigen in phosphate-buffered saline (PBS) by intravenous injection 3-4 days prior to fusion. Immunized spleen cells were fused with myeloma cells (P3X63 Ag8.653, ATCC, Rockville, MD). After 2 weeks, hybridoma supernatants were screened using a FMDV/O double antibody sandwich (DAS) ELISA. The positive clones were subcloned using a limiting dilution method. Isotyping was performed, using a mouse monoclonal antibody isotyping kit (Roche, Indianapolis, IN, USA).

### Purification of monoclonal antibody

Hybridomas were grown in BD Cell MAb medium (Becton Dickinson and company, MD, USA) supplemented with 2% FBS. After 7-9 days, the culture supernatants were harvested. The mAbs were purified from hybridoma culture supernatants by a HiTrap Protein-G affinity column (GE, Fairfield, CT) using an AKIA chromatography system according to manufacturer’s instruction.

### Peptides

A total of 41 and 43 overlapping peptides representing O1/BFS VP1 and VP2 (15 amino acids in length, overlapping each other by 10 amino acids) were synthesized by Mimotopes (Minneapolis, MN, USA). The peptide ELISA was performed according to the method described by Hohlich et al., [47].

### Indirect ELISA

Briefly, microtitre plates (Nunc Maxisorb, Roskilde, Denmark) were coated with purified virus or peptides or recombinant FMDV/O1/BFS VP1, VP2 [48]. After blocking, each hybridoma culture supernatant was added to platea. A horseradish peroxidase (HRP)-conjugated goat anti-mouse IgG (1:2000) was added, then O-phenylenediamine dihydrochloride (OPD, Sigma-Aldrich, St Lucia, MO, USA) was used for color development. The optical density (OD) was measured at 490 nm using an automated plate reader (Photometer Multiskan Reader, Labsystems, Foster, VA, USA). Each incubation step was 60 minutes at 37°C with gentle shaking and followed by washing three times with washing buffer (PBS with 0.05% Tween 20).

### Selection of mAb resistant mutants

FMDV O1/BFS1860 (TCID_50_ 10^7^/ml) was mixed with each purified neutralizing mAbs with dilutions 100-fold lower than minimum neutralization titer for 30 minutes at 37°C. The virus/mAb mixture and control without mAb were inoculated onto MVPK cells in T-25 flasks. The flasks were incubated at 37°C until 100% CPE observed. The culture supernatants were collected and clarified by centrifugation at 2000 *g* for 15 minutes. The 1 ml clarified supernatant was mixed with the respective mAb and inoculated onto MVPK cells for the next passage. The procedure was repeated six times. The mutants selected were purified by plaque purification.

### Two-dimensional virus neutralization test

A two-dimensional neutralisation test (2D-VNT), similar to that described by Booth et al. [49] was used. Briefly, two-fold serial dilutions of O1/Manisa vaccinated bovine serum provided by WRLFMD (our only available serum at the time) were reacted with 10^0^ to10^-3^ dilutions of virus for 1 hour at room temperature. MVPK cells were added and incubated at 37°C for 3 days. Antibody titres were calculated from regression data as the log10 reciprocal antibody dilution required for 50% neutralisation of 100 TCID50 of virus (log10 SN50/100 TCID50). The antigenic relationship of viruses based on their neutralisation by antibodies is given by the ratio: ‘r_1_’ = neutralising antibody titre of the heterologous virus/neutralising antibody titre of the homologous virus. Serological relationships between vaccine strain and field isolates in the range ‘r_1_’ = 0.3–1.0 are indicative of cross protection, whereas values < 0.3 indicate dissimilar vaccine strain and test strain [22].

### Antigenic profiling ELISA using mAbs

The antigenic profiling ELISA was performed as described previously [15,29]. Microtitre plates were coated with polyclonal rabbit anti-FMDV/O1 diluted in 0.06 M carbonate/bicarbonate buffer, pH 9.6 and incubated overnight at 4°C. The plates were blocked with a blocking buffer (5% skim milk in PBS with 0.05% Tween 20) at 37°C for 1 hour. The FMDV/O isolates diluted in blocking buffer were added to the plates. The panel of mAbs and a serotype independent mAb (F21-42) were added at optimal dilutions. After an incubation step, a HRP-goat anti-mouse IgG (1:2000) was added. Then a substrate OPD was used for color development. An equal volume of 2.0 M sulfuric acid was added to each well to stop the color reaction. The OD was measured at 490 nm using an automated plate reader.

To standardize the reading for the amount of the different virus attached to each well, the reactivity (R_X_) of each mAb with each isolate (X) was calculated based on the binding (OD) of a control mAb, F21-42 [18] by subtracting the respective blanks (B) using the formula R_X_ = [(O.D._mAbX_-O.D._mAbXB_)/(O.D. _Control mAb_-O.D._Control mAbB_) ×100]. The r values between O1/Manisa and each isolate were calculated based on the reactivity (Rx) obtained with each mAb as described by Seki [16].

### Sequencing FMDV O P1 gene

Genomic RNA was extracted from FMDV using the Rneasy Mini Kit (Qiagen). Terminal oligonucleotide primers (Invitrogen) complementary to the L gene (5’-TTCTGGTGTTTGTCCCGTACGAT-3’) and 2B gene (5’-GTTGACATGTCCTCCTGCATCTG-3’) for reverse transcription-PCR and additional ones for internal sequencing were chosen from the most conserved sequence region by alignments of available FMDV/O whole genome sequences from GenBank. Full-length cDNA copies of the P1 genes of each virus were synthesized from genomic RNA by using the terminal primers and SuperScript™ III reverse transcriptase (Invitrogen). The cDNAs were amplified by PCR using the Expand™ Long Template PCR system according to the manufacturer’s instruction (Roche). PCR products used for DNA sequencing were gel purified using QIAquick® gel extraction kit according to the manufacturer’s instruction (Qiagen). DNA sequencing was performed in both directions by use of an ABI Prism BigDye Terminator v3.1 Cycle Sequencing Ready Reaction kit (Applied Biosystems) and an Applied Biosystems Genetic Analyzer DNA Model 3130X. Sequences obtained from both directions were assembled and checked for accuracy with SeqMan® (Lasergene®, Version 9; DNASTAR, Inc.). Pairwise nucleotide sequence alignments were performed using the Martinez-NW method [50] and the Lipman-Pearson method [51] for protein alignments in MegAlign® (Lasergene).

### FMDV 3D structural analyses

Molecular graphics coordinates of the FMDV/O1/BFS1860 crystal structure (PDB # 1FOD) [52] were performed using the UCSF Chimera package from the Resource for Biocomputing, Visualization, and Informatics at the University of California, San Francisco [53]; supported by NIH (2P41RR001081). The resulting images were imported into Adobe Photoshop for editing.

## Competing interests

The authors declare that they have no competing interests.

## Authors’ contributions

MY: designed all experiments, produced and purified mAbs, performed peptide ELISA, antigenic profiling ELISA and drafted the manuscript. WX: performed 2D-VNT, sequencing and sequence analysis for FMDV isolates, identified mutations of selected mutants, and located mAb biding sites on the FMDV/O crystal structure. MG: performed mAb resistant mutant selection and plaque purification. ZZ: helped design 2D-VNT, provided the FMD viruses and vaccinated sera. WX and MG have read and approved the final version of the manuscript. ZZ is on leave of absence. All authors read and approved the final manuscript.
